# Protections of transcription factor BACH2 and natural product myricetin against pathological cardiac hypertrophy and dysfunction

**DOI:** 10.3389/fphys.2022.971424

**Published:** 2022-08-29

**Authors:** Xueli Jiang, Mengying Cao, Jian Wu, Xiaolin Wang, Guoping Zhang, Chunjie Yang, Pan Gao, Yunzeng Zou

**Affiliations:** Shanghai Institute of Cardiovascular Diseases, Zhongshan Hospital and Institute of Biomedical Sciences, Fudan University, Shanghai, China

**Keywords:** BACH21, cardiac hypertrophy2, heart failure3, myricetin4, AKAP65

## Abstract

Pathological hypertrophic myocardium under consistent adverse stimuli eventually can cause heart failure. This study aims to explore the role of BACH2, a member of the basic region leucine zipper transcription factor family, in cardiac hypertrophy and failure. Transverse aortic constriction surgery was operated to induce cardiac hypertrophy and failure in mice. BACH2 was overexpressed in mice through tail vein injection of AAV9-*Bach2*. Mice with systemic or cardiac-specific knockdown of *Bach2* were adopted. Neonatal rat ventricular myocytes (NRVMs) were isolated and infected with lentivirus to overexpress *Bach2* or transfected with siRNA to knock down *Bach2*. Our data showed that overexpression of BACH2 ameliorated TAC-induced cardiac hypertrophy and failure in mice and decreased isoproterenol (ISO)-triggered myocyte hypertrophy in NRVMs. Systemic or cardiac-specific knockdown of *Bach2* worsened the cardiac hypertrophy and failure phenotype in mice. Further assays showed that BACH2 bound to the promotor region of *Akap6* at the -600 to -587 site and repressed its expression, which functioned as a crucial scaffold for cardiac hypertrophy and failure signaling pathways. Small molecular natural product library screening suggested that myricetin could up-regulate expression of *Bach2* and simultaneously suppress the transcriptional levels of hypertrophic marker genes *Bnp* and *Myh7*. Further studies showed that myricetin exerted a BACH2-dependent protective effect against cardiac hypertrophy *in vivo* and *in vitro*. Taken together, our findings demonstrated that BACH2 plays a crucial role in the regulation of cardiac hypertrophy and failure and can be a potential therapeutic target in the future.

## Introductionis 

Cardiac hypertrophy in patients usually caused by hypertension or metabolic and genetic abnormalities (Maron et al., 2002; Shimizu and Minamino, 2016; Groenewegen et al., 2020). Clinical studies have shown that cardiac hypertrophy is a major cause of heart failure and cardiac death (Bos et al., 2009; Samak et al., 2016). The hypertrophic myocardium under consistent pathogenic stress eventually progresses into severe cardiac failure (Samak et al., 2016). To date, due to the incomplete understanding of the molecular mechanisms involved in cardiomyocyte hypertrophy, the existing therapeutic drugs are still very limited and less effective (Leache et al., 2021; Mohan et al., 2021). According to current guidelines, therapeutic strategies mainly focus on the treatment of complications and surgical resection in patients with severe hypertrophic obstructive cardiomyopathy (Maron et al., 2002; Bos et al., 2009). Therefore, it is critical to elucidate the molecular mechanism underlying the development of cardiomyocyte hypertrophy and thereby find effective pharmaceutical interventions.

Transcription factors Broad complex, tram track, bric-a-brac, Cap’n’Collar homolog one and 2 (BACH1 and BACH2) belong to the leucine zipper family member (Roychoudhuri et al., 2013; Kuwahara et al., 2014; Richer et al., 2016; Zhou et al., 2016). They form heterodimers with small Maf proteins or directly interact with other proteins at the BTB/POZ domain to suppress expression level of specific genes (Olivier Albagli et al., 1995). Of note, previous studies reported that depletion of BACH1 in mice protected against TAC-induced left ventricular remodeling and ischemia/reperfusion injury via up-regulating Heme Oxygenase-1 (Yano et al., 2006; Mito et al., 2008). BACH1 was also discovered to regulate the threshold of ferroptosis occurrence and the activation of nuclear factor (erythroid-derived 2)-like 2 (Nrf2) in related diseases including cardiac ischemia (Nishizawa et al., 2020; Sun et al., 2021). However, the role of BACH2 in cardiovascular diseases is still unclear.

This study aims to explore the detailed role and underlying mechanisms of BACH2 in cardiac hypertrophy and failure. To this end, the expression of BACH2 in human hypertrophic heart tissues and TAC-operated mice were detected. Mice with global or cardiac-specific knockout of *Bach2* as well as BACH2 overexpression by AAV9 were constructed to verify the function of BACH2 in cardiac hypertrophy and failure. In addition, natural product library was screened to find out the compound that may potentially target BACH2.

## Materials and methods

### Human heart samples

Human heart tissue slices were obtained at autopsy from Department of Forensic Medicine, Fudan University (approval No. 2019-023 and 2020-009). The hypertrophic group included those diagnosed with hypertension and cardiac hypertrophy (n = 7). The control group without previously diagnosed cardiovascular diseases was chosen from those died from car accident, poison or other non-cardiac diseases (n = 7). The relatives of all participants showed the full intention and signed the informed consents before the autopsy.

### Animals

All animal experiments were carried out according to the requirements of Animal Care Committee at Zhongshan Hospital, Fudan University. All mice were housed with ad libitum access to food and water in a temperature and light controlled cages (12h/12 h dark/light, 22 °C). During surgery and echocardiographic performance, mice were under general anesthesia induced by inhalation of 2.5-3% isoflurane and maintained with 2% isoflurane. The following mouse models were established:

1) **Conventional *Bach2* knockout mice:** Conventional *Bach2* knockout mice created by CRISPR/CAS9-mediated genome engineering in a C57BL/6J background were purchased from Cyagen Biosciences. The gRNA targets the sequence: ACA​GAC​GAA​AGA​TGA​CTT​GGT​GG (forward strand of gene), AGA​GAA​TGT​CCT​TCT​TCC​GTT​GG (reverse strand of gene). Due to the severe weight loss and high mortality rate of *Bach2* homozygous knockout mice after birth, *Bach2* heterozygous knockout (*Bach2* ^+/−^) mice were used in this study. Intragastric administration of myricetin (MCE, HY-15097) at a dose of 200 mg/kg/d was started the day after transverse aortic constriction operation.
2) **Cardiac-specific *Bach2* knockout mice:** Conditional cardiac-specific *Bach2* knockout mice were generated by crossing a-myosin heavy chain Mer-Cre-Mer transgenic mice with *Bach2* ^*fl/fl*^ mice. To induce *Bach2* knockout, tamoxifen was injected intraperitoneally at a dose of 40 mg/kg per day for three consecutive days. After 2 weeks recovery, TAC or sham operation was performed on these mice.
3) **Bach2 overexpression mice:** Adeno-associated virus nine vector (AAV9)- a myosin heavy chain-*Bach2* and the AAV9-*Gfp* control virus were constructed in HANBIO (Shanghai, China). C57BL mice aged 4 weeks old were purchased from Shanghai Jie Si Jie laboratory animal co. (Shanghai, China) and injected with AAV9-*αMhc*-*Bach2* or AAV9-*Gfp* through the tail vein. After 4-weeks vector expression, TAC or sham operation was performed on these mice.

### Transverse aortic constriction surgery

Background and age matched mice were subjected to TAC and sham operation. Mice were anesthetized by inhaling 2% isoflurane during the process. After mechanical ventilation and left thoracotomy, a 27-gauge needle (0.4 mm in diameter) was layed on the aortic arch located between brachiocephalic trunk and left common carotid artery. 6–0 nylon suture was used to ligate around the transverse aorta and needle, the needle was then removed to cause aortic stenosis. Except for the ligature, the sham surgery was operated in the same procedure.

### Echocardiographic analysis

After the mice were anesthetized with 2% isoflurane, echocardiographic analyses were performed with a VisualSonics Vevo2100 animal imaging instrument (VisualSonics, Inc.). M-mode images on parasternal left ventricular long axis were acquired to assess cardiac function and left ventricular wall thickness. Heart rate was above 400 bpm during the period of image acquisition.

### Isolation and culture of neonatal rat ventricular myocytes

Neonatal rat ventricular myocytes (NRVMs) were isolated from newly-born (1–2 days) Sprague-Dawley rats. The hearts were minced to 1 mm^3^ pieces and digested in 0.125% trypsin solution at 37°C. The supernatant was collected while the remaining tissues repeated this process for 4–6 times. The cell collections were passed through a 100 μm cell strainer and the tissue residues were filtered out. After cultivated in plates for 2 h with DMEM/F12 media containing 10% fetal bovine serum and 1% penicillin-streptomycin, the supernatant myocytes were collected for further study.

### *Bach2*-overexpressed lentivirus construction

The pLVX-*Bach2* expression plasmid encoding rat *Bach2* gene was acquired from MiaoLing Plasmid Sharing Platform (Wuhan, China). The package plasmids, psPAX2 and pMD2. G, were co-transfected with pLVX-*Bach2* expression plasmid in HEK 293T cell line to form *Bach2*-overexpressed lentivirus by the HieffTransTM Liposomal Transfection Reagent (YEASEN, Shanghai) according to the protocol. The culture media were collected and filtered by 0.45μm filter (Millipore, NY, USA), the supernatants of which were Bach2-overexpressed lentivirus and were added into neonatal rat ventricular myocytes together with polybrene (5 μg/ml).

### SiRNA transfection

The rat *Bach2* and *Akap6* siRNA were purchased from GenePharma (Shanghai, China). The si*Bach2* sequence was as below: sense: GCC​GGA​GAC​UCU​UUG​UAA​ATT, antisense: UUU​ACA​AAG​AGU​CUC​CGG​CTT. The si*Akap6* sequence: sense: CAA​ACG​ACC​UUG​AUC​AAG​ATT, antisense: UCU​UGA​UCA​AGG​UCG​UUU​GTT. SiPORT™ NeoFX™ Transfection Agent (Invitrogen) was used to transfect siRNA into NRVMs as instructed by users’ guide. Briefly, siPORT™ NeoFX™ was diluted and then incubated at room temperature for 10 min. Diluted RNA and diluted transfection agent were mixed and incubated for 10 min, and then dispensed into a culture plate overlayed with cell suspensions.

### Real-time quantitative PCR analysis

Trizol reagent (Vazyme) was used to extract total RNA from heart tissues or NRVMs according to the manual. Genomic DNA was then removed and cDNA was synthesized as instructed by Hifair^®^ III first Strand cDNA Synthesis SuperMix for qPCR (gDNA digester plus) (YEASEN, 11141ES10). Relative quantitative PCR was conducted in the Quantstudio six flex (Thermofisher) machine according to the instructions by SYBR Premix ExTaq kit (Cat#: RR420A, Takara, Japan). The forward and reverse primers were listed in Table 1.

**TABLE 1:** Primers of RT-qPCR.

	RT-qPCR: Forward primers	Reverse primers
*Anp* (Rat)	*ATC​ACC​AAG​GGC​TTC​TTC​CT*	*TGT​TGG​ACA​CCG​CAC​TGT​AT*
*Bnp*(Rat)	*AAG​ATG​GCA​CAT​AGT​TCA​AGC*	*AGA​AGA​GCC​GCA​GGC​AGA​GT*
*Myh7* (Rat)	*TGG​ATG​AGG​CAG​AGG​AGA​GG*	*TAG​GGT​TGG​GTA​GCA​CAA​GA*
*Bach2* (Rat)	*GAT​CAC​AGA​CCT​TCC​CAG​GA*	*TCT​CTT​TCT​CGC​ACA​CCA​GTT*
*β-actin* (Rat)	*CTG​TGC​CCA​TCT​ATG​AGG​GT*	*CTC​TCA​GCT​GTG​GTG​GTG​AA*
*Anp* (Mouse)	*GCT​TCC​AGG​CCA​TAT​TGG​AG*	*GGG​GGC​ATG​ACC​TCA​TCT​T*
*Bnp*(Mouse)	*GAG​GTC​ACT​CCT​ATC​CTC​TGG*	*GCC​ATT​TCC​TCC​GAC​TTT​TCT​C*
*Myh7*(Mouse)	*ACT​GTC​AAC​ACT​AAG​AGG​GTC​A*	*TTG​GAT​GAT​TTG​ATC​TTC​CAG​GG*
*Bach2*(Mouse)	*TCA​ATG​ACC​AAC​GGA​AGA​AGG*	*GTG​CTT​GCC​AGA​AGT​ATT​CAC​T*
*β-actin* (Mouse)	*GGC​TGT​ATT​CCC​CTC​CAT​CG*	*CCA​GTT​GGT​AAC​AAT​GCC​ATG​T*
*TGF-beta1*(Mouse)	*CTC​CCG​TGG​CTT​CTA​GTG​C*	*GCC​TTA​GTT​TGG​ACA​GGA​TCT​G*
*Fn1*(Mouse)	*GCT​CAG​CAA​ATC​GTG​CAG​C*	*CTA​GGT​AGG​TCC​GTT​CCC​ACT*
*Col1a1*(Mouse)	*GCT​CCT​CTT​AGG​GGC​CAC​T*	*CCA​CGT​CTC​ACC​ATT​GGG​G*
*Akap6* (Mouse)	GTG​GAT​GTG​CAT​CTA​GTT​CAG​C	CAT​TGA​CCG​AGT​AGG​ACA​GCA
*Akap6* (Rat)	CTA​GCA​CAT​CCC​AGT​TGC​CA	GCC​ACC​GTC​AAA​CCA​ACA​AA

### Western Blot

Tissues and cultured myocytes were lysed by RIPA buffer and then were centrifugated for 20 min at 4°C, 12000g to remove cell membranes. The supernatant was quantified by reacting with BCA working buffer (Thermo Fisher Scientific, Bremen, Germany). 12% or 8% SDS-PAGE gels were used to separate the target proteins, which was transferred to a PVDF membrane after wards. The membrane was blocked with 5% BSA buffer for 60 min at room temperature, followed by incubation with corresponding primary antibodies overnight at 4°C. After three washes with TBST buffer and incubated with horseradish peroxidase-conjugated secondary antibody, the bands were detected in LAS-3000 imaging system (FUJIFILM Inc. Tokyo, Japan). The antibodies used in this study were listed below: BACH2 (Affinity, DF2461; Cell Signaling, 80,775), β-actin (Cell Signaling, 3,700), α-actinin (Cell Signaling, 6,487), Myh7 (AB clonal, A7564). The original gel images were provided in the Supplementary Material.

### Immunofluorescence analysis

After washed with PBS three times, cultured NRVMs were fixed with 4% paraformaldehyde for 15 min. Then, the fixed NRVMs were washed again and permeabilizated with 0.5% Triton X-100 at room temperature for 20 min. Subsequently, the cells were blocked with 5% BSA for 30 min at room temperature and then incubated with anti-sarcomeric α-actinin antibody (abcam, ab9465) overnight at 4°C. The cells were washed three times and incubated with fluorescent secondary antibody for 1 h at room temperature. Afterwards, DAPI was added and incubated for 5–10 min. Last, the water was removed and the cells were sealed with antifade mountant.

### Cleavage under target & tagmentation and next-generation sequencing

NovoNGS^®^CUT&Tag 3.0 High-Sensitivity Kit (for Illumina^®^) was adopted in this study (Novoprotein Scientific Inc. Shanghai). Cultured AC16 cells in duplicate were collected in EP tubes and about 10^5^ cells were prepared for the continued experiment. Briefly, cells were firstly combined to the surface of concanavalin A (ConA) beads. Next, the cell membrane was permeabilized by digitonin and the ConA beads-cell combination was incubated with BACH2 primary antibody (Cell Signaling, 80,775) overnight at 4°C with rotation. The next day, the supernatant was separated and removed after the tubes were placed on magnetic frame for 2 minutes. Then, the goat anti-rabbit secondary antibody was added and incubated at room temperature for 1.5 h. Subsequently, Tn5 transposase was incubated and combined to the antibody-DNA complex. The DNA was then fragmented and extracted for the following DNA library construction and sequencing. The sequencing raw data were deposited in a public repository with an accession number of HRA002635 (https://bigd.big.ac.cn/gsa-human/browse/HRA002635).

### Chromatin immunoprecipitation assay

AC16 cells were cultured in DMEM media containing 10% FBS and 1% antibiotics. After cells reached 90% confluent, 1% formaldehyde was added to fix protein-DNA interactions and incubated for 10 min at room temperature. Next, glycine buffer was added to stop further crosslink. Cells were then lysed and chromatin was harvested and fragmented using both enzymatic digestion and sonication according to the instructions of SimpleChIP^®^ Enzymatic Chromatin IP Kit (Agarose Beads) (Cell Signaling Technology, U.S.A.). The DNA/protein fragments were subjected to immunoprecipitation using antibodies specific to BACH2. After immunoprecipitation, the protein-DNA cross-links were reversed and the DNA was purified for further RT-qPCR analysis. The *Akap6* promoter was detected using the forward primer 5′-GTA​GGC​GCA​AGA​AAT​GAG​CG-3′ and reverse primer 5′- TGG​GAA​CGA​AGC​AGA​GTC​AC-3’. The predicted BACH2 binding site on the promotor of *Akap6* was GGTGACTCTGCTTC based on the JASPAR database.

### Dual luciferase reporter gene assay

The day before transfection (12–24 h), HEK 293T cells were passaged at a density of 0.5-2 × 10^5^/well to a 96-well plate and cultured in DMEM media containing 10% FBS.

pGL3-*Akap6*wt promotor, pGL3-*Akap6*mut promotor, pEnCMV-*Bach2* and pEnCMV control plasmids at the quantity of 0.09 μg/well along with pGL4-renilla plasmid at 0.02 μg/well were transfected to the corresponding wells using HieffTransTM Liposomal Transfection Reagent (YEASEN, Shanghai). After cultured for 48 h, Dual luciferase Reporter Gene Assay (YEASEN, Shanghai) was performed in an opaque 96-well plate. The optical signal was detected using BioTeK hybrid reader.

### Histological analysis

The mice hearts were harvested and fixed immediately in 4% paraformaldehyde. They were then embedded in paraffin and cut into 5 μm slices. Hematoxylin and eosin (H&E) or wheat germ agglutinin (WGA) stain was performed in order to evaluate the cardiac size. Masson trichrome staining was carried out to calculate the cardiac fibrosis. For human left ventricle tissues stored in paraformaldehyde, we performed H&E, Masson and BACH2 immunohistochemistry stain for further analyses. The stained slices were then photographed and analyzed using the ImageJ software.

### Natural products screening and treatment

The extracted NRVMs were cultured in complete medium (DMEM/F12 + 10% FBS +1% Penicillin & Streptomycin) for 24 h. Then, the culture medium was replaced into FBS-free medium and NRVMs were starved for 12 h. Ninety-seven kinds of natural small molecule products (final concentration 2 μg/ml) were added to the corresponding treatment wells. One hour later, ISO diluted in PBS solution (final concentration 10 μΜ) was added to the compound-treated wells and ISO-treated alone wells. *In vitro* studies, myricetin was added 1 h preceded to 10 μM ISO (Meilunbio, Dalian) treatment at the indicated doses. In mice, the next day after surgical operation, WT and Bach2 heterozygous mice were treated with myricetin (200 mg/kg/d) or PBS control by gavage. The dosage of myricetin was adopted according to previous studies (Liao et al., 2017; Liao et al., 2019).

### Statistics

The results were presented as mean ± standard errors (SEM). For the data with homogenous variance and normal distribution, unpaired Student’s t tests were used to compare the difference between two groups and one-way ANOVA with Bonferroni post hoc test was applied to the comparisons among multiple groups. For the comparisons of multiple groups of data without equal standard deviation, Brown-Forsythe and Welch ANOVA tests were adopted. All statistical analyses were performed by GraphPad Prism 8 (GraphPad Software, Inc.). A *p* value < 0.05 was considered as statistically significant.

## Results

### Expression level of BACH2 in human and mouse hypertrophic hearts

**FIGURE 1:**
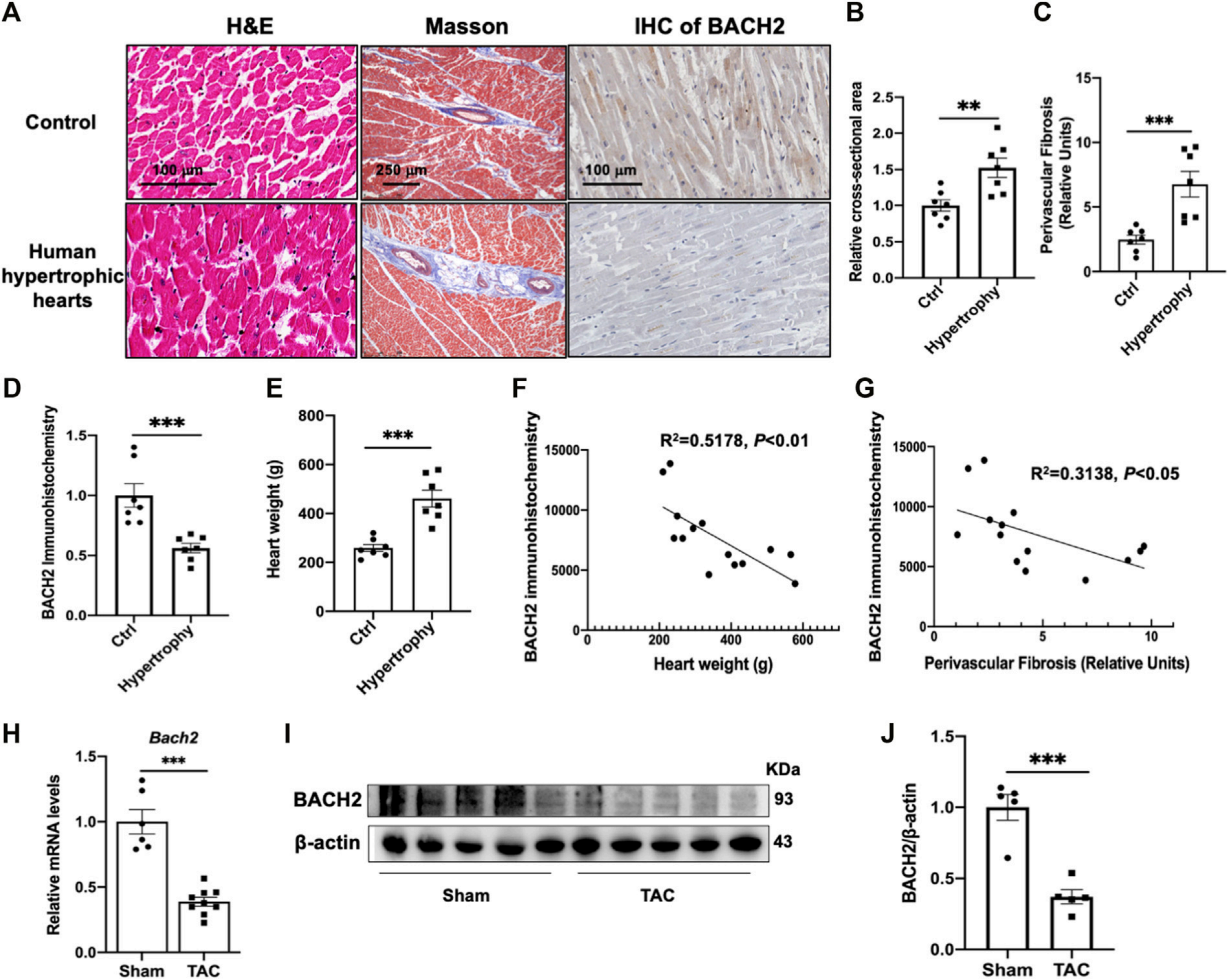
Expression of BACH2 in hypertrophic mouse hearts and histological analyses of human hypertrophic hearts. **(A)** H&E, Masson and immunohistochemistry (IHC) stain of human hypertrophic and control hearts (n=7). **(B–D)** Quantitative analyses of myocardial cross-sectional area, perivascular fibrosis (relative to vascular area) and BACH2 immunohistochemistry. Eight fields from each sample were randomly selected for analyses. **(E)** The heart weight difference. **(F–G)** The correlation of BACH2 immunohistochemistry quantitative value with heart weight and extent of perivascular fibrosis. (H) RT-qPCR analyses showing Bach2 mRNA levels in mouse hearts at 5 weeks after sham or transverse aortic constriction (TAC) surgery (n=6- 9). **(I–J)** Western blot and quantification analyses of BACH2 protein levels. *P<0.05, **P<0.01, ***P<0.001.

We first examined human hearts to determine whether the expression of BACH2 changed in hypertrophic hearts with pre-diagnosed hypertension compared with the control hearts without known cardiovascular diseases. H&E and Masson staining in heart tissue sections showed that cardiomyocyte cross-sectional area and perivascular fibrosis extent of hypertrophic hearts were larger than those of the control group (Figures 1A–C). Immunohistochemical staining of BACH2 revealed that the expression of BACH2 protein in hypertrophic hearts was significantly reduced than that in the control group (Figure 1D). The weight of hypertrophic hearts was significantly heavier than that of control hearts (Figure 1E). Further correlation analysis showed that the expression level of BACH2 was negatively correlated with heart weight and the extent of fibrosis (Figures 1F,G). These findings suggested that the expression of BACH2 in the heart was significantly downregulated during myocardial hypertrophy in human, the level of which was correlated with the degree of pathological progression. Next, we determined the expression level of BACH2 in mice hearts with TAC-induced cardiac hypertrophy and failure. Eight-week-old male wild-type C57 mice were subjected to TAC or sham operation. Five weeks later, the transcription levels of *Bach2* were significantly downregulated in TAC-operated mice compared to sham-operated animals (Figure 1H), and the protein expression levels of BACH2 were also decreased in TAC-operated mice compared to sham-operated ones (Figures 1I,J). These results indicated a loss of function of BACH2 during the development of cardiac hypertrophy and failure.

### Attenuation of cardiac hypertrophy by introduction of *Bach2* gene into cardiomyocytes

**FIGURE 2:**
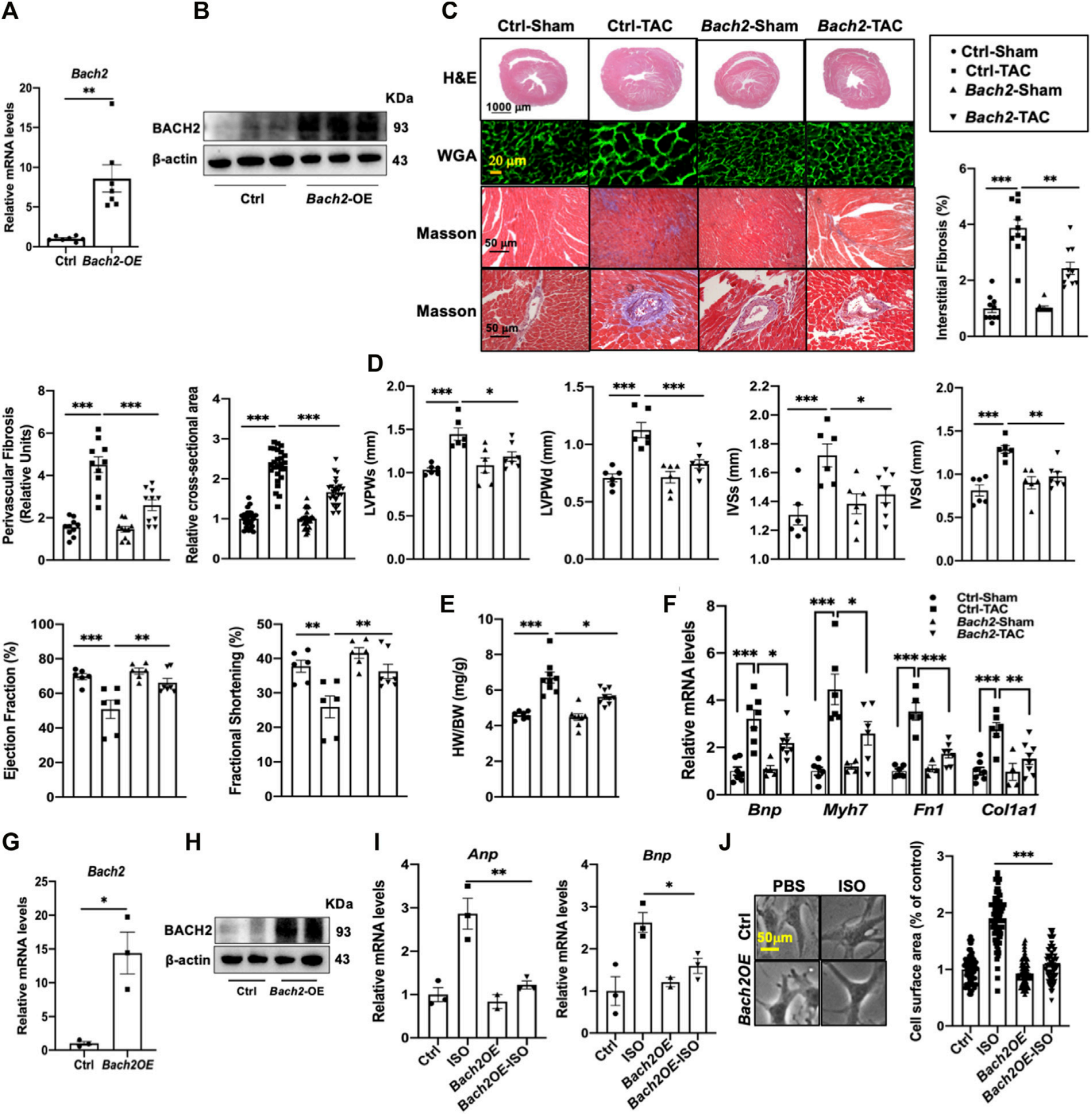
BACH2 overexpression attenuates cardiac hypertrophy and failure. **(A–B)** The relative *Bach2* mRNA and protein levels of mouse hearts injected with *Bach2* adeno-associated virus 9 (AAV9) or control virus through the tail vein. **(C)** Histological and quantification analyses of transverse heart sections. Hematoxylin-eosin (H&E) and wheat germ agglutinin (WGA) showed the hypertrophic cardiomyocytes. Masson stain demonstrated the perivascular (relative to vascular diameter) and interstitial fibrosis. **(D)** Echocardiographic analyses of cardiac systolic function and wall thickness. **(E)** Heart weight/body weight (HW/BW). **(F)** The mRNA levels of *Bnp, Myh 7, Col1a1* and *Fnl.* **(G–H)** NRVMs were transfected with lentivirus vector encoding rat *Bach2* or control lentivirus for 48 h. **(I)** Overexpression of BACH2 in NRVMs decreased the mRNA levels of *Anp and Bnp* after treated with 10 µM ISO for 24 h. **(J)** Overexpression of BACH2 decreased the cell surface area of NRVMs treated with IO µM ISO for 24 h photographed by bright-field microscope. *P<0.05, **P<0.01, ***P<0.001.

To define the functional role of BACH2 in regulation of cardiac hypertrophy and failure, we overexpressed BACH2 in TAC-induced cardiac hypertrophic mice with adeno-associated virus 9 (AAV9) encoding mouse *Bach2* by the tail vein injection. It was confirmed that *Bach2* transcription level in the myocardium of AAV9-*Bach2*-overexpression (OE) mice was approximately 8-fold higher than that of AAV9-control mice (Figure 2A). The BACH2 protein level in AAV9-*Bach2* mouse hearts was also significantly higher than that of AAV9-control ones (Figure 2B). 5 weeks after TAC surgery, the H&E and Masson staining revealed that the myocyte cross-sectional area and the extent of perivascular and interstitial fibrosis were decreased in *Bach2*-OE mice as compared to control mice (Figure 2C). Analysis of transthoracic echocardiography showed that ejection fraction and fractional shortening were improved in *Bach2*-OE mice but not in control group (Figure 2D). The left ventricular wall thicknesses referred as systolic or diastolic interventricular septum (IVSs and IVSd) and posterior wall (LVPWs and LVPWd) in *Bach2*-OE TAC mice were thinner than those in control TAC mice (Figure 2D). Compared with AAV9-control TAC mice, the heart weight/body weight ratio was significantly reduced in AAV9-*Bach2*-OE TAC mice (Figure 2E). Moreover, TAC-elicited increases in the expression of hypertrophic marker genes such as *Bnp* and *Myh7,* and fibrotic marker genes like type I collagen alpha 1 (*Col1a1*) and fibronectin 1 (*Fn 1*) were dramatically suppressed in *Bach2-*OE murine hearts (Figure 2F). These data demonstrated that the up-regulation of BACH2 resulted in a great regression of cardiac hypertrophy induced by pressure overload. We next examined the antihypertrophic effect of BACH2 in cultured NRVMs. Stimulation with ISO significantly induced hypertrophic responses characterized by increases in cell surface area and transcription levels of *Anp* and *Bnp*, whereas these hypertrophic responses induced by ISO were significantly alleviated in NRVMs infected with lentivirus encoding *Bach2* (Figures 2G–J). Both *in vivo* and *in vitro* data collectively indicated that overexpression of BACH2 played an inhibitory role in the development of cardiac hypertrophy.

### BACH2 downregulation exacerbates cardiac hypertrophy and failure

**FIGURE 3:**
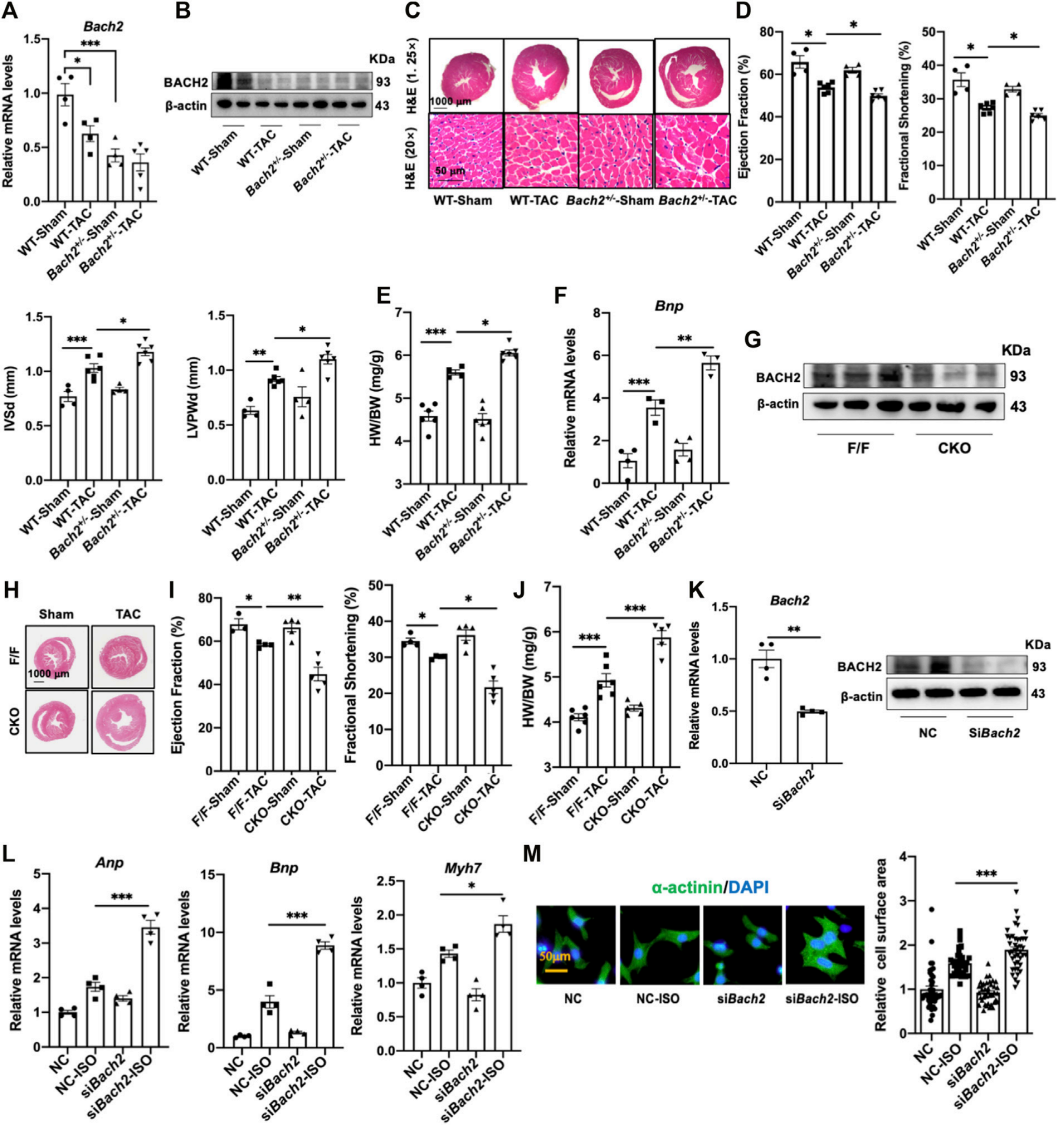
BACH2 deficiency exacerbates myocardial hypertrophy and dysfunction. **(A–B)** The *Bach2* mRNA and protein expression levels. **(C)** H&E stain showed the hypertrophic hearts. **(D)** Echocardiographic analyses of cardiac wall thickness and function. **(E)** Heart weight/Body weight (HW/BW). **(F)** RT-qPCR analyses of the *Bnp* mRNA expression level. **(G)** The protein expression level of BACH2 in conditional *Bach2* knockout mice compared with *Flox/F/ox* mice. **(H–J)** H&E stain, Heart weight/Body weight (HW/BW) and echocardiographic analyses of cardiac function. **(K)** The*Bach2* mRNA and protein levels in NRVMs after transfected with siRNA targeting Bach2 *(siBach2)* or negative control (NC). **(L)** RT-qPCR analyses showed that Bach2 silence decreased the mRNA levels of *Anp, Bnp* and *Myh7.* **(M)** Representative aαactinin staining immunofluorescence images of NRVMs treated with 10 µM ISO for 24h after transfected with *siBach2* or NC and quantitative analysis of cell surface area. *P < 0.05, **P < 0.01, ***P < 0.001.

Both systemic heterozygous *Bach2-*knockout mice and conditional cardiac specific *Bach2-*knockout mice were adopted in this study and subjected to TAC operation. When compared to wild type control mice, conventional heterozygous *Bach2-*knockout mice displayed a severer cardiac hypertrophy phenotype, such as more significant increases in HW/BW ratio, LV wall thickness and *Bnp* gene expression at 4–5 weeks after TAC (Figures 3A–F). Also, conditional cardiac specific *Bach2*-knockout mice convinced the same results as above, that is, higher HW/BW ratio and lower ejection fraction and fractional shortening than *Flox/Flox* control mice after TAC (Figures 3G–J). ISO-induced hypertrophic responses in NRVMs, such as increased cell surface area and reprogrammed gene expressions of *Anp*, *Bnp* and *Myh7* were all significantly enhanced when *Bach2 was knocked down* (Figures 3K–M). These *in vivo* and *in vitro* studies showed that depletion of BACH2 aggravated the progress of cardiac hypertrophy and failure.

### Identification of *Akap6* as a target gene of BACH2

**FIGURE 4:**
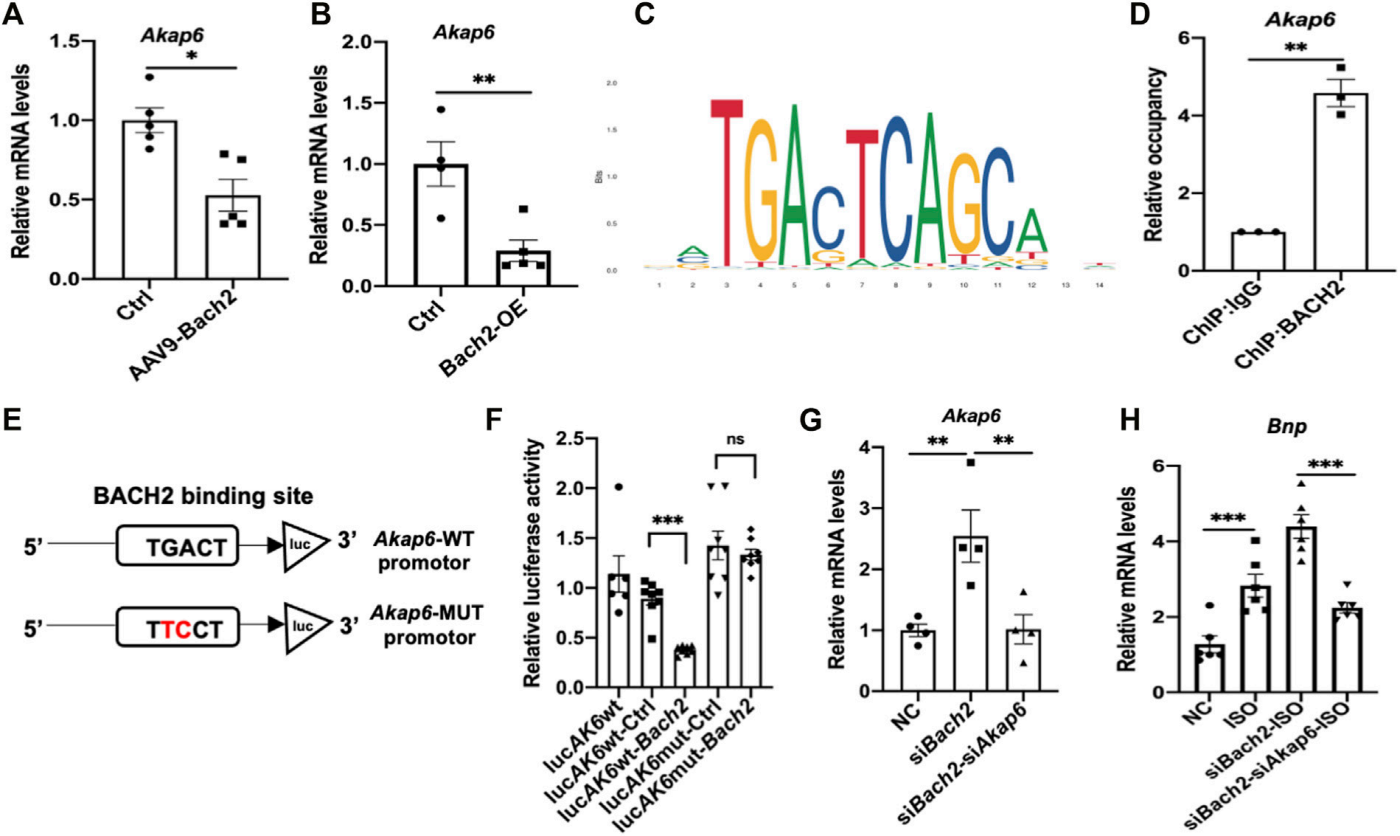
BACH2 regulates the expression level of *Akap6.* **(A)** The *Akap6* mRNA expression levels in Bach2 overexpressed and GFP control mice (n=5). **(B)** The *Akap6* mRNA expression levels in Bach2 overexpressed and control NRVMs. **(C)** Schematic diagrams showing the estimated binding site of BACH2 in *Akap6* promotor region based on the JASPAR database. **(D)** ChlP-qPCR analysis using ChIP-grade BACH2 antibody or IgG antibody to detect the binding of BACH2 to *Akap6* promotor. **(E–F)** Relative luciferase activity in HEK-293 cells transfected with reporter plasmids and Bach2 expression or control plasmid (n=8). **(G)** The mRNA expression levels of *Akap6.* **(H)** The hypertrophic marker gene *Bnp* expression levels (n=6). *P<0.05, **P<0.01, ***P<0.001.

Given the fact that BACH2 was identified as a transcription factor, we therefore tried to find its downstream target genes in cardiomyocytes. CUT&Tag experiment followed by next generation sequencing was carried out in this study. Among the screened genes, *Akap6* is well known for its role in cardiac hypertrophy and failure process. We then testified that the transcription level of *Akap6* was downregulated by BACH2 overexpression in mice hearts and NRVMs (Figures 4A,B). We searched JASPAR database for the putative binding site of BACH2 in the *Akap6* promotor region and the sequence “GGTGACTCTGCTTC” with the highest score at -600 to -587 site was highlighted (Figure 4C), which was confirmed by a following ChIP-qPCR analysis (Figure 4D). We next constructed an *Akap6* promotor mutant plasmid termed as *Akap6*-MUT by mutating the conservative sequence mentioned above (Figure 4E). The dual luciferase assay validated that although overexpression of BACH2 inhibited the activity of wild type *Akap6* promotor, it could not weaken the activity of mutant *Akap6* promotor (Figure 4F). Further study in NRVMs showed that *Bach2* knockdown induced the increase in the transcription level of *Akap6* (Figure 4G), and *Akap6* silencing blunted the effect of *Bach2* knockdown alone in enhancing the expression level of hypertrophic marker gene *Bnp* under ISO stimulation (Figure 4H). These data collectively suggested that BACH2 protected against cardiomyocyte hypertrophy and dysfunction via targeting *Akap6*.

### Natural product myricetin exerted a BACH2-dependent protective effect against cardiac hypertrophy and failure

**FIGURE 5:**
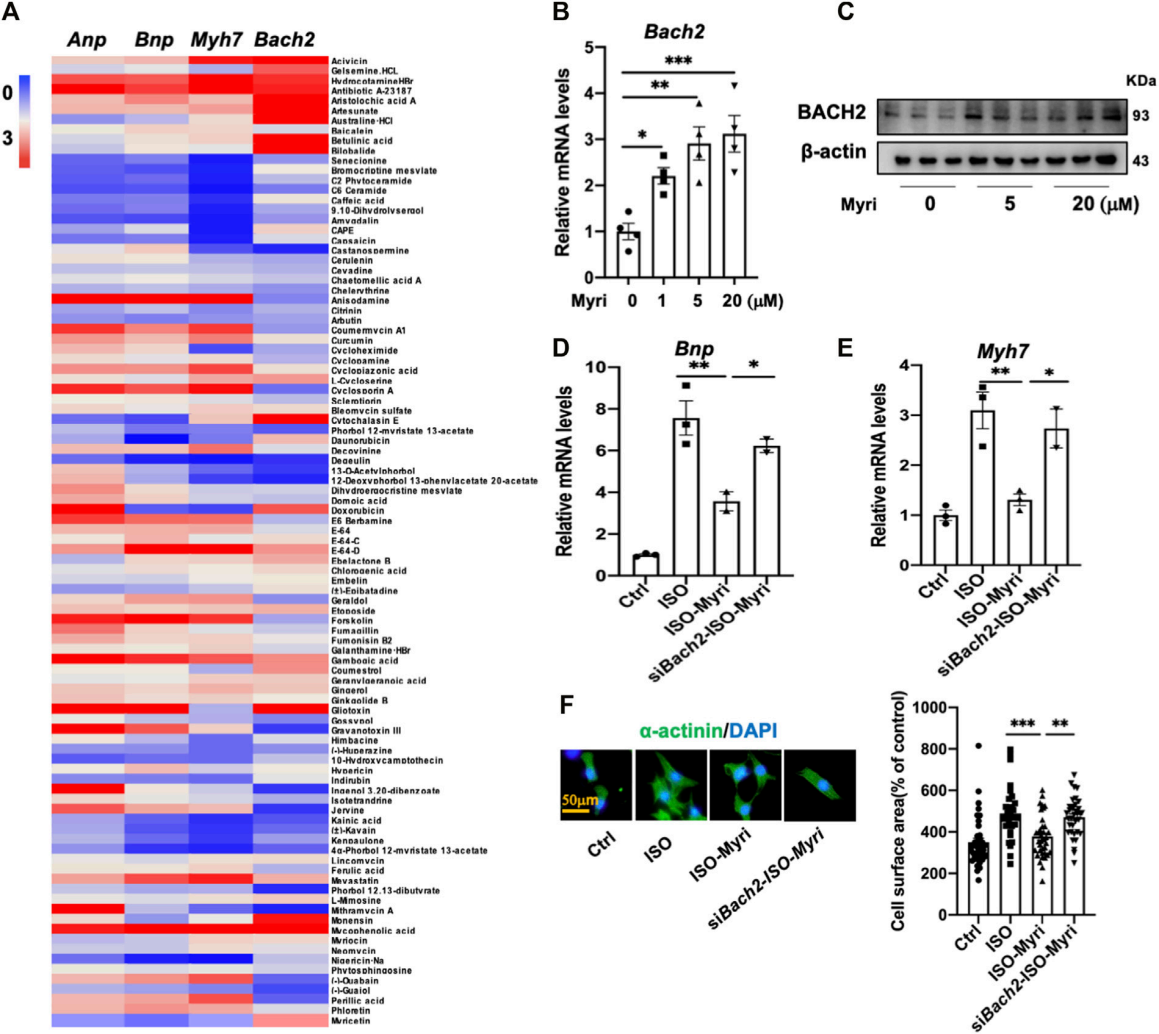
Myricetin attenuates myocyte hypertrophy partially via up-regulating BACH2 expression levels *in vitro.* **(A)** Heat map of the relative *Anp, Bnp, Myh 7* and *Bach2* mRNA levels in the NRVMs treated with both ISO and natural products compared to the group treated with only 10 µM ISO. **(B)** The *Bach2* mRNA levels in indicated NRVMs (n=4). **(C)** Western blot showed the relative protein levels of BACH2 in the NRVMs exposed to *5* and 20 µM myricetin (n=3). **(D–E)** Myricetin inhibited ISO-induced increased hypertrophic gene expression levels, whereas Bach2 deficiency blocked this effect. **(F)** Representative a-actinin staining immunofluorescence images of NRVMs treated with 10 µM ISO and 20 µM myricetin for 24 h after transfected with *siBach2* or NC and quantitative analysis of cell surface area. *P<0.05, **P<0.01, ***P<0.001.

**FIGURE 6:**
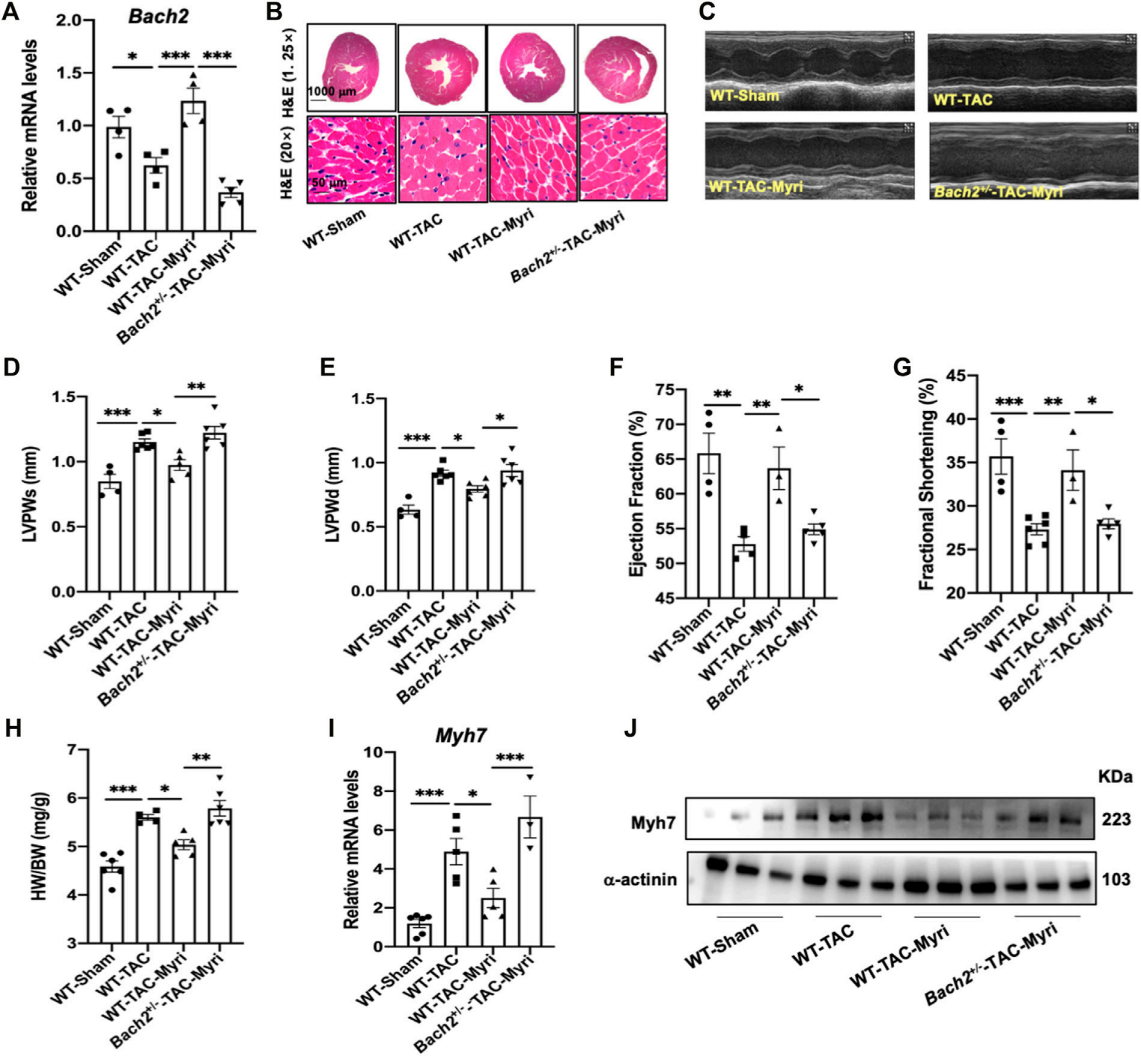
Myricetin attenuates TAC-induced cardiac hypertrophy and failure partially via upregulating BACH2 expression levels *in vivo.* **(A)** *Bach2* transcriptional levels in the indicated groups of mice (n=4-6). **(B)** Representative H&E images showed heart transverse sections. **(C)** The representative M­ mode echocardiographic images. **(D–G)** Echocardiographic indicators showed that myricetin treatment alleviated TAC-induced wall thickening and cardiac dysfunction whereas this effect was blocked in the *Bachr1·mice*. **(H)** Heart weight/Body weight (HW/BW). **(l–J)** The mRNA and protein level of *Myh 7* in the indicated groups. *P<0.05, **P<0.01, ***P<0.001.

By RT-qPCR analyses of 97 kinds of natural products in NRVMs, myricetin was identified as the most potent one to upregulate the transcription level of *Bach2* and decrease the expression of hypertrophic marker genes under the stimulation of ISO (Figures 5A–C). To testify whether the protective effect of myricetin against cardiomyocyte hypertrophy is dependent on BACH2, cultured NRVMs were transfected with siRNA targeting *Bach2*, pretreated with myricetin and then exposed to ISO. Although myricetin suppressed ISO-induced cardiomyocyte hypertrophic responses, such as *Bnp* and *Myh7* gene expression and increased cell surface area, these protective effects of myricetin were attenuated by *Bach2* silencing (Figures 5D–F). Finally, we determined the therapeutic effect of myricetin on TAC-induced cardiac hypertrophy in mice. Wild type and *Bach2* heterozygous knockout mice received TAC or sham operation were administered myricetin (200 mg/kg/day) or vehicle by gavage. Myricetin administration dramatically elevated *Bach2* expression level in TAC-operated wild type mice (Figure 6A). Moreover, the evaluation of HW to BW ratio, H&E staining of heart tissue and echocardiography analysis showed that myricetin treatment alleviated these indexes of cardiac hypertrophy and dysfunction induced by TAC in wild type mice. However, these effects of myricetin were attenuated in *Bach2* heterozygous knockout mice (Figures 6B–H). In addition, the upregulated mRNA and protein expression level of *Myh7* induced by TAC was blocked by myricetin treatment in wild type mice but not in *Bach2* heterozygous knockout mice (Figures 6I,J). Taken together, these results showed that myricetin exerted a BACH2-dependent protective effect against cardiac hypertrophy and failure.

## Discussion

In this study, we found that the expression level of BACH2 was significantly downregulated in mouse hypertrophic hearts induced by TAC. Overexpression of BACH2 protected the mice from TAC-induced cardiac hypertrophy and failure. Systemic or cardiac specific knockdown of *Bach2* worsened the cardiac hypertrophy and failure phenotype in mice after TAC. Meanwhile, BACH2 overexpression in NRVMs alleviated ISO-induced myocyte hypertrophy and its deficiency aggravated this phenotype. To elucidate the potential mechanism underlining the role of BACH2 in cardiac hypertrophy and failure, our study showed that BACH2 bound to the promotor region of *Akap6* at the -600 to -587 site and repressed its expression. AKAP6 is a well-known master scaffold protein that orchestrates cardiac hypertrophy and heart failure signaling. Moreover, our current study showed that natural product myricetin ameliorated cardiac hypertrophy and failure partially via up-regulating BACH2.

Previous studies reported that BACH2 as a basic leucine zipper family transcription factor regulated differentiation, infiltration, accumulation and activation of immune cells. However, the role of BACH2 in cardiac hypertrophy and failure is still unknown. In this study, we found that the expression of BACH2 was remarkably downregulated in hypertrophic hearts of murine and humans, suggesting a potential role of BACH2 in the regulation of cardiac hypertrophy and failure. It is known that the formation of cardiac hypertrophy is due to the disequilibrium between pro-hypertrophic and anti-hypertrophic factors. We used myocardium-specific AAV9 vector to overexpress BACH2 in mouse hearts and lentivirus vectors encoding *Bach2* to infect cultured cardiomyocytes. On the other hand, heterozygous *Bach2* knockout mice and conditional cardiac specific *Bach2* knockdown mice were constructed. Both *in vivo* and *in vitro* results showed that upregulation of BACH2 significantly inhibited the development of cardiac hypertrophy, whereas BACH2 knockdown exacerbated the progression of cardiac hypertrophy. These results indicate that BACH2 functions as an anti-hypertrophic molecule in cardiomyocytes.

BACH2 as a transcription factor activates or represses the expression level of its target genes (Oyake et al., 1996). Therefore, to explore the potential mechanism underlying the anti-hypertrophic effect of BACH2, CUT&Tag technology followed by next generation sequencing was conducted. We searched the literature and discovered that among the dozens of genes filtered out, *Akap6* is a well-studied gene in cardiomyocytes encoding the protein that participates in cardiac hypertrophy process. Our study further suggested that BACH2 bound to the -600 to -587 site of the *Akap6* promotor region and overexpression of BACH2 downregulated the transcription level of *Akap6*. We also observed in our study that knockdown of *Akap6* in cardiomyocytes with siRNA could block BACH2 deficiency-caused aggravation of hypertrophic responses. The protein AKAP6, located mainly in the nuclear membrane of cardiomyocytes, acts as a scaffold of signaling complexes that contribute to cardiac hypertrophy development and progress (Pare et al., 2005; Kimura et al., 2010; Li J. L et al., 2013; Kritzer et al., 2014; Passariello et al., 2015). AKAP6 integrates multiple signaling pathways involved in the induction and formation of cardiac hypertrophy and failure which is induced by diverse malignant stimuli including cytokines, growth factors and pressure overload. Not only as a cAMP-dependent protein kinase binding protein, AKAP6 binds to a variety of myocyte remodeling response proteins including PLCε, nesprin-1α, adenylyl cyclase type 5, exchange protein activated by cAMP-1 (Epac1), cAMP-specific phosphodiesterase type 4D3, MEK5 and ERK5 MAP-kinases, 3-phosphoinositide-dependent protein kinase-1, p90 ribosomal S6 kinases 3 (RSK3), protein kinase Cε, protein kinase D, Ca2^+^/calmodulin-dependent phosphatase Aβ(CaNAβ), and so on. Through binding with PDK1, AKAP6 activates MAPK-RSK3 signaling. RSK3-AKAP6 anchor disruption or interfering RSK3 expression alleviates hypertrophy of NRVMs. RSK3 depletion in mice attenuates pressure overload or catecholamine induced cardiac hypertrophy (Li J et al., 2013). The AKAP6 binding protein PLCε is also required for *in vitro* NRVMs hypertrophy and *in vivo* TAC-induced cardiac hypertrophy (Zhang et al., 2013). AKAP6 is also necessary for activation of PKD and phosphorylation of HDAC4 in response to pressure overload (Kritzer et al., 2014). The binding of CaNAβwith AKAP6 influences Ca^2+^ release in myocytes and is required for the formation of pathological cardiac hypertrophy (Li et al., 2010). In addition, in TAC mice AKAP6 regulates the activation of NFATc, MEF2 and HDAC4 (Wilkins et al., 2002; Bourajjaj et al., 2008; Kim et al., 2008; Li J L et al., 2013). In brief, the modulation of multiple cardiac hypertrophy and failure signaling pathways was mediated by AKAP6 scaffold protein. Previous studies found that in NRVMs AKAP6 was required for α- and β-adrenergic hormones induced myocyte hypertrophy. Moreover, it has been demonstrated that cardiomyocyte-specific knockout of *Akap6* in mice or knockdown of *Akap6* by siRNA in cardiomyocytes has protective effects against corresponding TAC-induced cardiac hypertrophy and heart failure or ISO-triggered cardiomyocyte hypertrophy (Pare et al., 2005; Kritzer et al., 2014). These studies suggested that the role of transcription factor BACH2 in cardiac hypertrophy and failure could be mediated by AKAP6.

Given the results of the inhibitory effect of BACH2 overexpression on cardiac hypertrophy, we tried to screen natural products that could up-regulate BACH2 expression. Among the detected 97 compounds selected from the natural products library, myricetin was found to significantly upregulate the expression of BACH2 and simultaneously suppress hypertrophic responses of ISO-stimulated cardiomyocytes. Myricetin is known to be a flavonoid compound widely present in plants and daily food (Wang et al., 2019). It has been shown that myricetin has beneficial effects in a variety of cardiovascular diseases, including myocardial infarction, ischemia-reperfusion injury, heart failure and myocardial injury caused by lipopolysaccharide, endotoxin and ISO (Cominacini et al., 2015; Gupta et al., 2020; Taheri et al., 2020; Zhang et al., 2020; Song et al., 2021). In consistent with these reports, our present study showed that myricetin dramatically improved cardiac hypertrophy and failure. Previous studies suggested that myricetin enhanced the expression and activity of Nuclear factor erythroid-related factor 2 (Nrf2), the Cap-n-collar basic-region leucine zipper family transcription factor, which also binds to the Maf recognition element of target genes (Liao et al., 2017; Liao et al., 2019). We here found that myricetin could inhibit cardiac hypertrophy in wild type mice and cardiomyocytes, but not in Bach2-deficient hearts and cardiomyocytes. Taken together, these data showed that the mechanisms of the protective role of myricetin in cardiovascular diseases are multifaceted. However, the mechanism underlying how myricetin upregulates BACH2 expression requires further study.

In conclusion, our study demonstrates that BACH2 plays an important role in the regulation of cardiac hypertrophy and failure*.* For the treatment of clinical cardiac hypertrophy and heart failure, BACH2 may serve as a potential valuable intervention target in the future.

## Data Availability

The sequencing raw data were deposited in the Genome Sequence Archive with accession number HRA002635 (https://bigd.big.ac.cn/gsa-human/browse/HRA002635).

## Abbreviations

AAV9, adeno-associated virus 9; ANP, atrial natriuretic peptide; BNP, brain natriuretic peptide; CUT&Tag, cleavage under target & tagmentation; ChIP, chromatin immunoprecipitation; H&E, hematoxylin and eosin; ISO, isoproterenol; Myh7, myosin heavy chain beta; NRVMs, neonatal rat ventricular myocytes; RT-qPCR, real-time quantitative PCR; TAC, transverse aortic constriction; WGA, wheat germ agglutinin.
