# Supplementary figures and images for: Protections of transcription factor BACH2 and natural product myricetin against pathological cardiac hypertrophy and dysfunction
Source: Front Physiol. 2022 Aug 29;13:971424. doi: 10.3389/fphys.2022.971424 (PMC9465486; doi:10.3389/fphys.2022.971424)

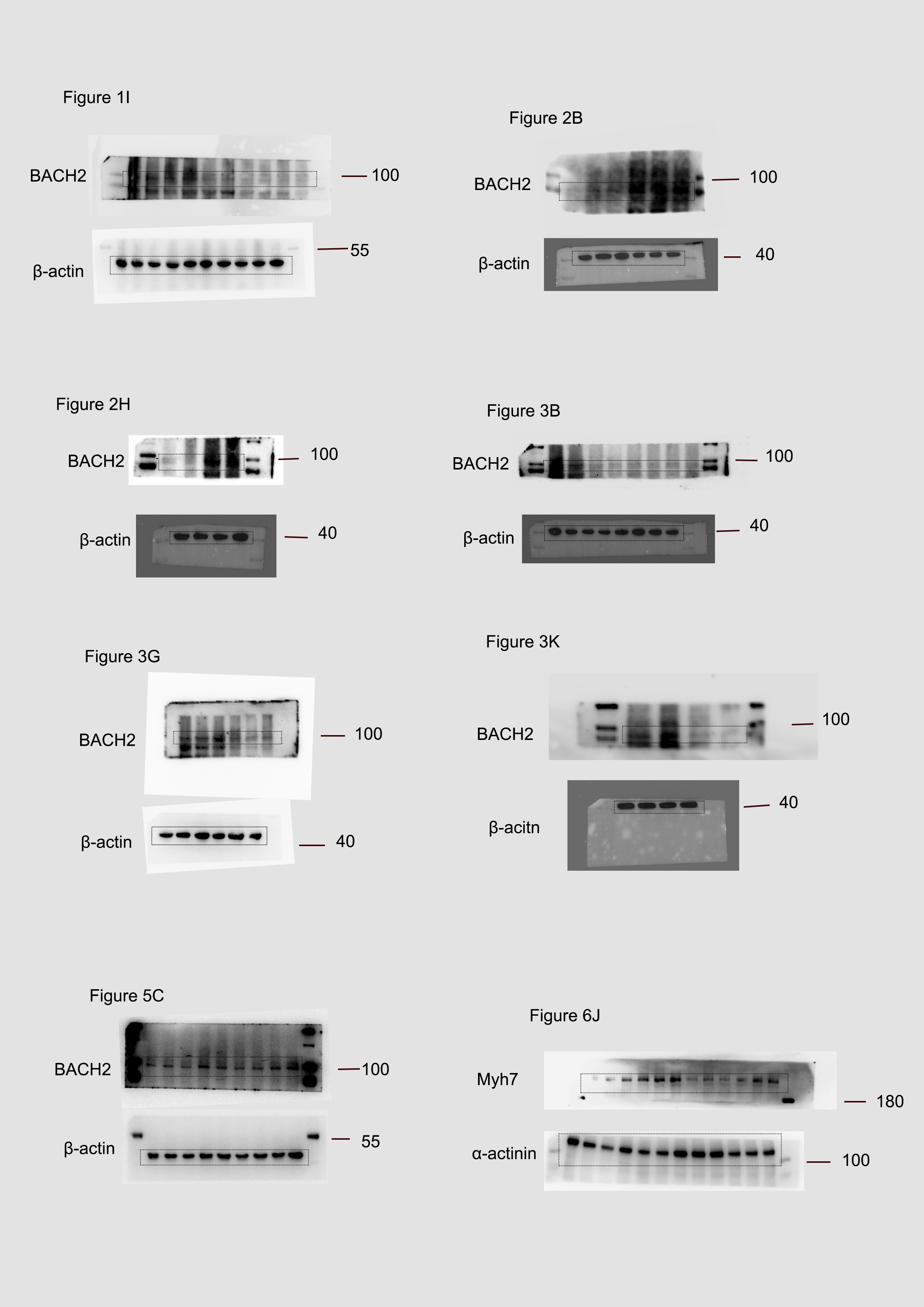

Supplement: Supplementary file 1 [file Image1.jpg]
